# Comparison of long-term outcomes between Off-Pump CABG and conventional CABG

**DOI:** 10.1186/1749-8090-10-S1-A243

**Published:** 2015-12-16

**Authors:** Won Yong Lee, Jung Hyun Lim, Kun IL Kim, Hyoung Soo Kim

**Affiliations:** 1 Cardiothoracic Surgery, Hallym University Sacred Heart Hospital, Anyang, 431-070, South Korea; 2 Cardiothoracic Surgery, Chosun University Hospital, Gwangju, 501-717, South Korea; 3 Cardiothoracic Surgery, Hallym University Kangnam Sacred Heart Hospital, 150-950, South Korea

## Background/Introduction

There has been controversy surrounding the late outcomes of Off-Pump Coronary Artery Bypass (OPCAB).

## Aims/Objectives

The aim of this study was to compare the early and long-term outcomes of OPCAB with those of conventional CABG (c-CABG).

## Method

This retrospective study was based on data from 183 patients who underwent CABG between January, 2000 and December, 2005. OPCAB was performed in 102 patients and c-CABG in 81. The mean follow-up duration was 107 months. The end-points of long-term results were overall death, freedom from cardiac death and major adverse cardiac events (MACE). We expressed the Kaplan-Meyer survival curve, and determined the independent predictors for risk factors of mortality using multi-variate analysis.

## Results

Four patients in c-CABG group died of low cardiac output syndrome and CVA. There was no operative mortalities in OPCAB group (p = .023). Bleeding requiring reopening (5:1, p = .05) and CVA (3:0, p = .05) occurred more frequently in c-CABG group than OPCAB group. The completeness of follow-up was 83.8%.

Late deaths occurred in 26 patients (11 [18.0%] in c-CABG group, 15 [16.9%] in OPCAB group). The causes of death were cancer, CVA, cardiac and sepsis.

Rerevascularization was performed more frequently in OPCAB group than in c-CABG group (14:5, p = .297). Five-year overall survival, freedom from cardiac death and MACE in c-CABG and OPCAB groups were 90.2 vs 96.6 %, 98.4 vs 100 %, and 91.8 vs 85.4 %, respectively. Ten-year survival estimates were 82.0 vs 83.1 %, 96.7 vs 96.6 %, and 88.5 vs 83.1 %, respectively. There were no statistical significance between 2 groups' survival (p = .743 in overall survival, P = .813 in free from cardiac death, and p = .305 in free from MACE). Age was an independent predictor for mortalities (p = 0.000).

## Discussion/Conclusion

OPCAB showed the better operative mortality and complication rates, and the higher rerevascularization rate, compared with c-CABG. Nevertheless, the survival indices did not reveal the statistical significance between 2 groups.

